# Low NIHSS score in large vessel occlusion stroke: optimal treatment and clinical controversies

**DOI:** 10.3389/fneur.2025.1681311

**Published:** 2025-11-10

**Authors:** Tonghe Chen, Wenhong Zhi, Ning Hao, Zaili Li, Xu Cao, Qiuchi Chen, Li Zhang, Zhiguang Liu

**Affiliations:** 1 Department of Neurology, XuZhou Clinical School of XuZhou Medical University, Xuzhou, Jiangsu, China; 2 Department of Neurology, Southeast University Affiliated Xuzhou Central Hospital, Xuzhou, Jiangsu, China; 3 Department of Cardiology, Dangshan County People’s Hospital, Dangshan, Anhui, China

**Keywords:** low NIHSS score, large vessel occlusion, endovascular thrombectomy, early neurological deterioration, clinical controversies, individualized treatment

## Abstract

Acute ischemic stroke caused by large vessel occlusion (LVO) with low National Institutes of Health Stroke Scale (NIHSS) scores (≤5) presents a critical clinical dilemma regarding optimal management. While endovascular thrombectomy (EVT) is established for moderate-to-severe strokes, its role in milder cases remains controversial, balancing potential benefits against risks of intracranial hemorrhage and procedural complications. This review synthesizes evidence from observational studies, registry data, and meta-analyses comparing EVT with best medical therapy (including intravenous thrombolysis and antiplatelet treatment) in this population. Key findings indicate no significant difference in 90-day functional outcomes between EVT and medical management; across observational cohorts, EVT has been associated with higher symptomatic intracranial hemorrhage (sICH) and a possible increase in 90-day mortality, but these estimates derive from non-randomized data and may reflect selection bias and residual confounding. Subgroup analyses highlight the influence of occlusion location (proximal vs. distal), risk of early neurological deterioration (END), time window, and bridging therapy on treatment decisions: proximal occlusions (e.g., internal carotid artery, middle cerebral artery M1 segment) and high END risk may favor more aggressive intervention, while distal occlusions (e.g., M2 segment) often respond adequately to medical therapy with close monitoring. Clinical recommendations emphasize an individualized approach: prioritizing medical management for most patients, with EVT reserved for high-risk cases or those with neurological deterioration during observation. Future randomized controlled trials are needed to refine patient selection criteria and validate risk stratification tools for this challenging population.

## 1 Introduction

Acute ischemic stroke due to large-vessel occlusion (LVO) is typically a neurologic emergency where rapid recanalization via endovascular thrombectomy (EVT) significantly improves outcomes in patients with moderate-to-severe deficits (1). However, up to half of all ischemic strokes present with relatively mild symptoms (NIHSS ≤5) (2), approximately 4–11% of these mild strokes harbor a large-vessel occlusion [population-based registry: 4.0% (3); national registry: 8.1% (4); hospital cohorts with routine vascular imaging: ~10.6% (5)]. The optimal management of LVO stroke patients with low NIHSS is controversial (6). On one hand, even minor deficits can become disabling (e.g., isolated aphasia or hemianopia), and there is a risk of early neurological deterioration (END) if the occlusion persists (5). On the other hand, urgent EVT carries procedural risks that might outweigh its benefit if the patient’s deficits would remain mild or spontaneously improve (7). Clinical practice varies widely: some centers favor immediate intervention for any LVO, whereas others adopt a conservative approach with medical management and close observation (6). Current guidelines reflect this uncertainty. Intravenous thrombolysis (IVT) is generally recommended for disabling strokes regardless of NIHSS (8), however IVT may be withheld in non-disabling minor strokes due to unclear benefit–risk balance (9). For EVT, American and European guidelines do not endorse routine thrombectomy in NIHSS≤5 outside of clinical trials, instead advising case-by-case judgment or enrollment in ongoing studies (8). Consistent with this uncertainty, randomized evidence specifically enrolling low-NIHSS LVO patients remains limited; consequently, much of the current literature relies on retrospective observational studies and registry data. This narrative review will explore the controversy surrounding EVT versus best medical therapy (BMT)—which includes IV thrombolysis or antiplatelet management—in LVO patients presenting with low NIHSS. We summarize current evidence from observational studies, registries, and meta-analyses comparing outcomes of EVT and medical therapy, and discuss subgroup considerations (occlusion location, early deterioration, time window, and use of bridging thrombolysis) that inform clinical decision-making. We also propose an evidence-based approach for managing these patients and highlight directions for future research.

## 2 Search strategy and selection criteria

We conducted a targeted narrative literature search of PubMed/MEDLINE, Embase, Web of Science, and the Cochrane Library (inception to October 2025). Search strings combined stroke-specific and population terms (e.g., “large vessel occlusion” OR LVO) AND (NIHSS OR “mild stroke” OR “NIHSS ≤5”) AND (thrombectomy OR “endovascular therapy” OR EVT) with modifiers for occlusion site/time window (M1/M2/ICA; “0–6 h”; “6–24 h”; “early neurological deterioration”). We included English-language human studies enrolling adult patients and reporting management or outcomes of low-NIHSS LVO, as well as guidelines and meta-analyses. We excluded pediatric studies, non-LVO minor stroke cohorts, conference abstracts without full texts, and single-patient case reports (unless used for definitions). Two reviewers independently screened records and extracted study characteristics and outcomes (90-day mRS, sICH, mortality; subgroup data by time window and occlusion site). Because this is an evidence synthesis with heterogeneous, mostly observational data, we did not conduct a PRISMA-style systematic review or formal risk-of-bias assessment; instead, we provide a qualitative appraisal and narrative synthesis emphasizing study design and confounding.

## 3 Controversies in managing low-NIHSS LVO stroke

### 3.1 Uncertain benefit of EVT in mild strokes

A major controversy is whether EVT confers enough benefit in mild LVO strokes to justify its risks. Initial EVT trials mostly excluded NIHSS<6 (1), so direct evidence is lacking. Retrospective studies have yielded conflicting results. Some single-center series suggest EVT can improve outcomes even in NIHSS≤5 patients (10). For example, Yedavalli et al. conducted a retrospective cohort study including 46 patients with minor stroke and M1/M2 occlusions (11 treated with thrombectomy and 35 managed medically) and reported that those treated with thrombectomy had significantly better 90-day outcomes (median modified Rankin Scale (mRS) 1 vs. 3) (10). In that study, EVT led to a greater shift toward neurological improvement and functional independence (10). This implies that for certain “mild” LVO strokes, reperfusion therapy may prevent disability that would manifest despite an initially low NIHSS.

However, larger registry-based analyses have found no clear functional advantage to immediate EVT over medical management in this population (6). In a matched comparison of 544 patients (NIHSS ≤5) from the German Stroke Registry vs. SITS-ISTR registry, Matusevicius et al. observed no significant difference in 3-month good outcome between those who underwent EVT (± IVT) and those who received IVT alone (77.0% vs. 82.9%, *p* = 0.119) (6). Symptomatic ICH and mortality rates were similar between groups as well (6). These findings suggest many low-NIHSS LVO patients do well with medical therapy alone. The authors concluded that IV thrombolysis alone was as effective as EVT (with or without IVT) in minor LVO strokes, calling for randomized trials for definitive guidance (6).

A 2024 meta-analysis of 22 observational studies (patients treated 2015–2023) found no difference in functional outcomes between EVT and medical management, but higher odds of sICH (OR≈3.36) and a possible increase in 90-day mortality (OR≈1.84) with EVT. These non-randomized data are vulnerable to selection bias and residual confounding. In contrast, a propensity-matched registry analysis of NIHSS≤5 LVO reported similar mortality between EVT ± IVT and IVT-only groups. Overall, any mortality difference remains uncertain (7). Thus, there is genuine controversy: while EVT reliably improves recanalization, in mild strokes this may not always translate to better disability outcomes, and the intervention itself carries non-negligible risks (7).

### 3.2 Which patients to treat?

Clinicians struggle to identify which “mild” LVO patients actually stand to benefit from intervention. A low NIHSS can be misleading—some patients have mild scores yet significant disability (e.g., isolated cortical deficits) (11), whereas others truly have small infarcts and will do well without intervention (12). Because treatment recommendations often hinge on whether symptoms are disabling, we make this explicit with an operational, context-dependent definition (see Box 1) (13).

BOX 1 **Operational definition of “disabling deficit” (context-dependent)**

Definition: a disabling deficit is a neurological impairment that, if unchanged, would (a) prevent the patient from performing basic activities of daily living (ADLs) or (b) preclude return to work/usual roles. This ADL/return-to-work standard is the convention used in major guidelines and trials to distinguish “mild but disabling” from “mild, non-disabling.” (8).

NIHSS-anchored examples commonly regarded as disabling (TREAT Task Force). Complete hemianopia (NIHSS Q3 ≥ 2); severe aphasia (Q9 ≥ 2); visual or sensory neglect (Q11 ≥ 1); any limb weakness that limits sustained effort against gravity (Q5 or Q6 ≥ 2); any other deficit judged disabling by the clinician and the patient (e.g., dominant-hand fine-motor loss in a musician, communication-critical dysarthria) (14).

Note: guidelines recommend IV alteplase for mild but disabling symptoms and advise against thrombolysis for mild, non-disabling minor stroke; therefore, explicitly classifying disability is pivotal (13).

Table 1 summarizes the major recommendations of the AHA/ASA and ESO guidelines regarding IVT and EVT in low-NIHSS LVO patients, highlighting areas of consensus and controversy.

**Table 1:** Comparison of AHA/ASA (2019) and ESO (2019/2021) guidelines on management of low-NIHSS (≤5) LVO Stroke.

Aspect	AHA/ASA (2019)	ESO (2021 IVT & 2019 EVT)
IV thrombolysis (IVT)	Recommended for eligible patients with NIHSS≤5 if the deficit is disabling (e.g., causes functional impairment); do not withhold alteplase solely due to low NIHSS when symptoms are potentially disabling. IVT is not recommended for truly non-disabling mild strokes (NIHSS ≤5 with no significant functional deficit) (13)	Recommended for low-NIHSS strokes if deficits are disabling, similar to AHA (29). In minor non-disabling strokes, IVT is generally discouraged; however, if a large-vessel occlusion is present, ESO expert consensus *still suggests giving IV thrombolysis* despite mild non-disabling symptoms (acknowledging limited evidence) (29). This nuanced stance reflects concern for early deterioration in LVO.
Endovascular thrombectomy (EVT)	Not routinely recommended for NIHSS≤5 LVO strokes. The AHA/ASA guidelines did not include low-NIHSS patients in the proven EVT criteria, so mechanical thrombectomy is not standard for NIHSS ≤5 (29). Benefit in this group is unproven—EVT may only be considered in carefully selected cases (e.g., truly disabling deficits) on an individualized, case-by-case basis (Class IIb, evidence uncertain) (13).	No routine EVT for low-NIHSS LVO—instead, strongly encourage enrollment in clinical trials for this population (62). The ESO advises that if NIHSS≤5 LVO patients cannot be randomized in a trial, EVT can be considered only if there are clearly disabling deficits or if the patient clinically worsens despite IVT (62). No recommendation to perform EVT in purely non-disabling mild LVO strokes (no consensus among experts to treat those) (62).
“Mild but disabling” symptoms	Recognized and emphasized. AHA/ASA explicitly defines “mild stroke with disabling symptoms” as a scenario where the NIHSS is low but the neurologic deficit would impair the patient’s normal life (e.g., affects activities of daily living or ability to work) (13). Such deficits—for example, isolated but significant weakness, aphasia, or visual field loss—are considered “disabling” mild strokes and warrant full treatment (IVT, and evaluation for EVT per physician judgement).	Recognized similarly. ESO guidelines note that even low NIHSS strokes can have disabling deficits (common examples: aphasia, significant motor weakness, or hemianopia) that are not reflected in the raw score. These patients are treated as having meaningful deficits—i.e. they should receive IVT, and EVT should be considered if other factors favor it. Mild deficits deemed non-disabling (e.g., very minor sensory symptoms) are distinguished from disabling ones and are generally managed conservatively (62).
Individualized decision-making	Yes—case-by-case. The AHA/ASA encourages individualized clinical judgment for low-NIHSS LVO cases. Given the lack of definitive trial data, treatment decisions should be tailored: reperfusion therapy *may be reasonable* in select patients if potential benefits outweigh risks (13). The guideline’s Class IIb recommendation for thrombectomy in low-NIHSS reflects this cautious, individualized approach.	Yes—case-by-case. ESO guidelines explicitly support an individualized approach when evidence is sparse. For a low-NIHSS LVO patient not in a trial, the stroke team should weigh factors (age, deficit severity, occlusion characteristics, infarct risk) and make a case-by-case decision (62). In practice, this means closely assessing if a “mild” LVO patient has high risk of deterioration or truly disabling deficits—those patients may be treated more aggressively, whereas others are observed.
Trial inclusion or observation	Observation (with medical management) is the default if no treatment is given. The AHA/ASA guidelines do not explicitly call for trial enrollment in this group, but they acknowledge uncertain benefit and thus implicitly endorse conservative management for low-NIHSS LVO strokes not meeting treatment criteria (13). Such patients are typically managed with best medical therapy (e.g., antiplatelets) and monitored for any neurological change.	Trial enrollment is encouraged. ESO guidelines recommend enrolling low-NIHSS LVO patients in clinical trials whenever possible to gather evidence (62). If trial inclusion is not available, a strategy of close observation with planned intervention is favored over passive watchful waiting in high-risk cases (29). ESO experts advise against a “wait and see” approach if the patient has factors predicting deterioration—instead, be prepared to proceed with thrombectomy at the earliest sign of worsening. For stable truly mild cases, careful observation is used, but the importance of trials (e.g., *ENDOLOW*, *MOSTE*) is underscored to guide future practice.

*AHA/ASA, American Heart Association/American Stroke Association (2019 update of the guidelines for the early management of acute ischemic stroke); ESO, European Stroke Organisation (2019 ESO-ESMINT guideline on mechanical thrombectomy; 2021 ESO guideline on intravenous thrombolysis). NIHSS, National Institutes of Health Stroke Scale; LVO, large vessel occlusion; IVT, intravenous thrombolysis; EVT, endovascular thrombectomy.

When an intracranial LVO coexists with disabling deficits (Box 1), we consider the patient functionally “non-minor” despite a low NIHSS and frame reperfusion decisions accordingly (13).

Furthermore, a subset of patients initially mild will neurologically deteriorate if the occlusion is not opened (15). Approximately 12–30% of minor stroke LVO patients experience early neurological deterioration (for the purpose of this review, END primarily refers to a worsening of ≥4 points on the NIHSS within 24 h of presentation or treatment, consistent with common usage in acute ischemic stroke studies) due to extension of the infarct or inadequate collateral flow (16). These patients often end up with poor outcomes if managed medically (17). The dilemma is that performing immediate EVT on everyone would overtreat the 70–88% who might survive without deficit, yet treating no one would undertreat the high-risk subset that will worsen. Identifying predictive factors for END is an area of active research (12). Factors like occlusion location and clot characteristics have emerged as important: proximal occlusions (ICA or proximal M1) and longer thrombus length correlate with higher odds of END in low-NIHSS strokes (12). A simple ENDi risk score based on occlusion site and thrombus length has been retrospectively derived and externally validated in minor-stroke LVO patients, showing moderate discrimination (C-statistic ≈0.76–0.78). However, it lacks prospective validation and no prespecified thresholds have been linked to treatment decisions. Notably, perfusion/collateral metrics (e.g., HIR) were not associated with ENDi in that cohort and were not included in the score (12). For now, this score should be viewed as a risk-stratification tool that may assist decision-making rather than a therapy-guiding instrument (12). Absent a perfect predictive tool, strategies range between two extremes—“treat upfront” vs. “wait-and-see.” This controversy underlies much of the discussion on subgroups below. Endovascular thrombectomy (EVT) for posterior-circulation low-NIHSS LVO remains uncertain for two reasons. First, the NIHSS underestimates many brainstem signs (e.g., gaze palsy, dysarthria, ataxia), so a “low” score may not equal a minor posterior-circulation deficit (18). Second, the pivotal basilar-artery occlusion (BAO) RCTs that demonstrated benefit largely enrolled moderate–severe strokes—ATTENTION (NIHSS ≥ 10) and BAOCHE (NIHSS ≥ 6) showed superiority of EVT over medical therapy, whereas BASICS (which included milder BAO) was neutral overall; consequently, extrapolation to very-low NIHSS BAO is limited (19). An intention-to-treat meta-analysis pooling BEST/BASICS/ATTENTION/BAOCHE confirmed overall benefit of EVT in BAO, but no treatment effect was observed in the NIHSS < 10 subgroup (underpowered), aligning with the clinical impression that patients with higher baseline NIHSS derive clearer benefit (20). For posterior cerebral artery (PCA) occlusion—especially P1, often presenting with low NIHSS—comparative data remain observational and mixed. The multicenter PLATO registry (*n* = 724) found no functional advantage of EVT ± IVT versus IVT alone (adjusted common OR 1.07, 95% CI 0.79–1.43) but higher odds of sICH (aOR 2.87, 95% CI 1.23–6.72) and mortality (aOR 1.77, 95% CI 1.07–2.95); early neurological improvement was more frequent with EVT (21). The TOPMOST case–control study in distal PCA (P2–P3) suggested feasibility/safety yet no clear superiority over medical therapy (22). Overall, these data support a cautious, individualized approach: EVT is reasonable for BAO when deficits are at least moderate and imaging is favorable, whereas for very-low NIHSS BAO or PCA-P1 the routine use of EVT is not clearly supported; decisions should hinge on disabling symptoms and imaging-based risk (proximal/flow-limiting location, perfusion mismatch) (23).

### 3.3 Role of thrombolysis vs. antiplatelets

Another debated point is the best medical management for these patients if EVT is not performed. Standard IV thrombolysis is approved regardless of NIHSS for any disabling deficits, but many minor LVO strokes are initially labeled “non-disabling,” leading some physicians to forgo thrombolysis. The PRISMS trial (minor stroke without LVO) suggested no big benefit of tPA over aspirin in NIHSS<5 non-disabling strokes (24), but LVO-positive patients are a different cohort. Registry data indicate that giving IV tPA to minor LVO strokes may improve outcomes (25). In the SITS registry, IVT (versus no reperfusion treatment) independently predicted good 3-month outcome in low-NIHSS LVO patients (OR ~2.16) (6). Thrombolysis may also reduce the chance of END by partially recanalizing the occlusion or improving collaterals (12). In contrast, for patients who truly have tiny infarcts and robust collaterals, immediate treatment even with tPA might expose them to hemorrhage risk for little gain (26). In the ARAMIS randomized trial of patients with minor, non-disabling acute ischemic stroke within 4.5 h, dual antiplatelet therapy (clopidogrel + aspirin) was non-inferior to intravenous alteplase for excellent 90-day outcomes, with fewer early neurologic worsening/bleeding events in the alteplase arm (27). A recent meta-analysis of randomized trials in minor stroke (NIHSS ≤5) found that IV thrombolysis did not improve 90-day functional outcomes versus non-thrombolytic standard care and was associated with higher risks of sICH and mortality, underscoring ongoing uncertainty in this population (28). However, in the specific setting of LVO, most experts still favor IVT (if no contraindications) over antiplatelets alone, given the occlusion present (29). The controversy thus is mainly about EVT on top of medical therapy, rather than thrombolysis versus antiplatelets, and recent data continue to weigh against routine EVT in this group absent clear indicators of potential deterioration.

In summary, the key controversies boil down to patient selection and timing: Should we intervene early in all LVO with NIHSS≤5, or only treat those who worsen? Is it ever justified to do nothing (no tPA, no EVT) initially? The following sections examine evidence from subgroup analyses that attempt to clarify these questions.

## 4 Subgroup analyses and key factors

### 4.1 Occlusion site: M1 vs. M2 vs. ICA

Occlusion location appears to significantly influence the risk–benefit profile in low-NIHSS strokes. Proximal large vessel occlusions (terminal ICA or proximal M1 segment of MCA) occlude a large territory, yet occasionally present with mild symptoms due to good collateral circulation (30). These cases have a high risk of secondary deterioration if collaterals fail. In a study of minor strokes after IVT, isolated ICA occlusion was strongly associated with early neurological worsening despite thrombolysis (16). Many stroke experts thus view ICA occlusions with NIHSS≤5 as ticking time bombs—given the large brain region at risk, some advocate for urgent thrombectomy even if deficits seem mild, especially if the patient is clinically frail or has subtle signs that could become disabling. In contrast, M2 branch occlusions involve smaller territories (31); in clinical practice, a mild deficit with an M2 occlusion might remain mild. Data support this distinction. An analysis by Seners et al. found that the impact of adding EVT (“bridging therapy”) in minor stroke varied by occlusion site: in M1 occlusions, bridging therapy was associated with higher odds of excellent outcome compared to IVT alone (OR 3.26 for proximal M1) (32). By contrast, in M2 occlusions, bridging therapy was actually associated with lower odds of excellent outcome (OR 0.53), suggesting IVT alone fared better (32). This implies that for M1 occlusions, upfront EVT can rescue tissue that tPA alone often cannot (due to larger clot burden), whereas for M2 occlusions, the added benefit of thrombectomy is low and may be outweighed by procedural harm.

In minor stroke with isolated M2 occlusion, a multicenter matched analysis by Alexandre et al. found no difference in 90-day outcomes or safety between early mechanical thrombectomy (eMT) and best medical management with optional rescue thrombectomy (rMT); about one-quarter of medically managed patients required rMT after early neurological worsening, yet a selective “rescue-only” strategy yielded outcomes comparable to treating everyone upfront, supporting an initial medical approach with rescue for early deterioration (33). Importantly, within M2 occlusions, it is important to distinguish dominant/proximal M2 (larger-caliber branch supplying a substantial portion of the MCA territory or a proximal M2 origin) from non-dominant/distal branches (34). Observational cohorts suggest that EVT for dominant/proximal M2 can achieve M1-like functional outcomes with acceptable safety in carefully selected patients, whereas for non-dominant/distal M2—often grouped under DMVO—randomized data remain sparse and the net benefit of EVT is uncertain; IV thrombolysis should be prioritized when eligible, and EVT decisions individualized (ideally within trials) (34). Current AHA/ASA guidance states that EVT may be reasonable for selected M2 occlusions (Class IIb, Level B-R) (13).

For M1 occlusions, the calculus may differ. While no randomized data exist, the evidence above suggests proximal M1 (and likely carotid-T or ICA) occlusions are more likely to benefit from intervention (35). If a patient with an ICA or M1 occlusion has any hint of disabling deficit (even if NIHSS is 3–5) or if advanced imaging shows a large perfusion territory at risk, many stroke centers will proceed with EVT. Conversely, an M2 occlusion patient with NIHSS 1–2 who is truly asymptomatic or very mild can often be observed (with IVT if eligible) given the lower risk territory. In summary, location matters: treat proximal occlusions more aggressively, whereas distal occlusions (especially M2 inferior division or beyond) can often be managed conservatively at first. This nuance is a key factor in individualized decision-making.

### 4.2 Early neurological deterioration (END)

The occurrence (or high risk) of early neurological deterioration is a pivotal factor guiding treatment in mild LVO stroke (12). Currently, there is no unified standard definition for END, which generally refers to a significant deterioration in neurological function from the baseline NIHSS score in patients during the early period (typically within 24–72 h) after the onset of acute ischemic stroke. END is observed in a significant minority of these patients and portends worse outcomes (15). In one multicenter cohort, roughly 1 in 4 patients with an initially minor LVO stroke deteriorated within 24 h when managed medically, usually due to infarct progression (33). A multicenter JNIS study of minor-stroke patients undergoing thrombectomy reported ~25% END, which independently predicted worse 90-day outcomes; unsuccessful recanalization and post-procedural ICH were associated with END (36). Identifying predictors of END can help target EVT to those who need it most. As noted, occlusion site (ICA or proximal MCA) is one predictor (12). Thrombus length or burden is another—longer clots are less likely to lyse spontaneously and more likely to cause worsening (37). Poor collateral blood flow on imaging is also associated with END (though collateral status may be indirectly tied to occlusion site and clot extent) (38).

One study proposed a scoring system (the END score) incorporating occlusion site (ICA/M1 vs. M2), thrombus burden, and collateral status to predict deterioration risk (12). Patients with high risk might be triaged to immediate EVT despite low NIHSS. In practice, even without a formal score, stroke teams often assess factors like CT angiography clot burden and collateral grade. For example, a patient with NIHSS 2 from an ICA occlusion, negligible collaterals, and a long clot would be deemed very high risk for END—many would argue for urgent EVT in this scenario to preempt a likely decline. On the other hand, a patient with NIHSS 4 from an M2 occlusion with excellent collaterals might be safely observed.

Importantly, if a patient is managed conservatively initially, close neurological monitoring is essential to catch early deterioration. Upon the emergence of early neurological deterioration (END), immediate consideration should be given to “salvage” thrombectomy, provided that the intervention remains within a viable time window (17). Many centers admit these patients to intensive monitoring and serial NIHSS checks. The threshold for “rescue” thrombectomy is typically any significant worsening or new deficit emergence. There is evidence that performing EVT at the first sign of neurologic decline can still lead to good outcomes (39). However, one must be cautious—once severe deficits develop, some damage is done (40). The challenge is not intervening too late. Some experts advocate a very low threshold to escalate to thrombectomy, given that once NIHSS increases, outcomes tend to worsen. The interplay of END risk has also been studied in analysis of EVT efficacy: a recent meta-analysis noted that none of the existing retrospective studies selectively targeted high-END-risk patients for EVT (41). This suggests current data (showing no overall EVT benefit) might underestimate EVT’s value in a subset who were most at risk of END. Future trials may incorporate an END-risk stratification to clarify if targeted EVT is beneficial. For now, the presence or likelihood of END is a critical factor—if high, one leans toward intervention; if low, one can justify medical management.

### 4.3 Time window: early (0–6 h) vs. late (6–24 h) presentation

Time from stroke onset is another important consideration. All the above-discussed studies predominantly included patients in the early time window (mostly 0–6 h from onset for EVT or 0–4.5 h for IVT), because standard acute therapies are typically given in that period. For late-presenting patients (6–24 h) with LVO and mild deficits, the approach is even less defined (42).

Randomized late-window trials (DAWN and DEFUSE-3) demonstrated the benefit of EVT when patients are selected by advanced imaging (clinical-core mismatch or target perfusion mismatch), but both trials excluded patients with NIHSS <6; therefore, no RCT-level evidence exists for mild strokes in the 6–24-h window (43). A recent multinational CLEAR subanalysis in Neurology (*n* = 318 low-NIHSS [≤5] anterior-circulation LVO, 6–24 h) found no difference in 90-day disability outcomes between EVT and medical management; symptomatic ICH and mortality were also not significantly different (Class III evidence) (44). Observational data suggest feasibility of imaging-selected late-window EVT in mild deficits. In a multicenter Chinese cohort of mild ischemic stroke (NIHSS 0–8) with LVO, patients who underwent EVT 6–24 h with a perfusion/DWI mismatch had similar 90-day outcomes and safety to those treated ≤6 h, indicating that delayed EVT can be performed safely when a substantial mismatch is present; however, this study did not compare against best medical management (45). Case-based literature also documents successful “very-late” salvage guided by mismatch (e.g., EVT ~ 52 h after last-known-well), underscoring that imaging-based selection can identify patients with persistent penumbra despite mild symptoms (46). Analyses around the late-window trials further inform borderline NIHSS ranges: a DEFUSE-3 secondary analysis of NIHSS 6–9 (i.e., just above the “mild” threshold) suggested a trend toward benefit with EVT in the 6–16-h window, although not statistically significant within that small subgroup (47). Outside strict perfusion-software selection, late-window EVT guided by computed tomographic angiography (CTA)-based collaterals also appears effective (e.g., MR CLEAN-LATE), but these studies predominantly enrolled NIHSS ≥6 and cannot be extrapolated wholesale to NIHSS ≤5 (42). On how to select low-NIHSS patients in the late window, most groups pragmatically borrow the DEFUSE-3 target mismatch thresholds (core <70 mL, mismatch ratio ≥1.8, and absolute mismatch ≥15 mL) or DAWN’s clinical-core paradigms, and then layer clinical risk (proximal ICA/M1 occlusion, poor collaterals, longer clot, or evolving deficits). In such patients, off-label EVT may be reasonable on a case-by-case basis, particularly when the deficit is actually disabling or when END risk is judged high—while acknowledging the lack of RCTs specific to NIHSS ≤5. Current expert guidance (e.g., SVIN late-window statement) supports imaging-based selection in late windows, but stops short of recommending routine EVT for NIHSS ≤5 (48). Preferred practical framing for late-window, low-NIHSS LVO. For patients with NIHSS ≤5 presenting at 6–24 h, immediate EVT may be reasonable only when advanced imaging demonstrates a robust mismatch (e.g., DEFUSE-3–like profile) and there is proximal occlusion (ICA/M1) or poor collaterals/substantial clot burden, or when the deficit is clearly disabling or early neurological deterioration is emerging. Otherwise, best medical therapy with close monitoring and rescue EVT upon deterioration is appropriate, acknowledging the absence of RCTs dedicated to NIHSS ≤5 (48). At present, there is no randomized trial dedicated exclusively to late-window (6–24 h) low-NIHSS LVO, and available evidence remains observational (44).

Generally, for a minor LVO stroke in the 6–24 h window, clinicians will either observe or, if available, enroll the patient in a trial (several ongoing RCTs like ENDOLOW are investigating EVT in low NIHSS including late window). The extended window patients who remain mild likely have good collaterals (49), which could sustain the penumbra for some time—this argues for a conservative approach unless deterioration occurs. Additionally, after 6 h, IV tPA is usually not an option [except in wake-up stroke with favorable MRI, but those again usually require a notable deficit to justify thrombolysis (50)]. Thus, beyond 6 h, the “best medical therapy” is generally antithrombotic (aspirin or DAPT) and observation, EVT may be performed in select cases with clear radiographic jeopardy or if any clinical worsening ensues. Neither AHA/ASA nor ESO provides a distinct antithrombotic protocol for “mild LVO,” so treatment follows minor-stroke/TIA pathways (51). For minor non-cardioembolic stroke/TIA (typically NIHSS ≤3 in trials), short-term dual antiplatelet therapy (DAPT) lowers early recurrence at the cost of a small increase in bleeding, as shown in CHANCE (aspirin–clopidogrel for 21 days), POINT (aspirin–clopidogrel), and THALES (aspirin–ticagrelor for 30 days) (52). Accordingly, when IV thrombolysis is not given, many experts employ a brief course of DAPT in low-NIHSS LVO managed medically—most commonly aspirin plus clopidogrel for ~21 days, then de-escalation to single antiplatelet therapy to limit bleeding—consistent with AHA/ASA and ESO guidance and pooled analyses showing benefit concentrated in the first 2–3 weeks (51). Use of glycoprotein IIb/IIIa inhibitors (e.g., tirofiban) is not recommended routinely outside clinical trials because efficacy in acute ischemic stroke is unproven and bleeding risk is a concern (13). Neuroprotective agents are not routinely recommended by AHA/ASA or ESO (Class III: No Benefit), although regional guidelines vary (e.g., Japan supports edaravone [Grade B] and Chinese guidelines allow individualized use of brain-cell–protection agents); ongoing RCTs (e.g., NBP, edaravone–dexborneol) show signals but have not yet changed international guidance (13). In summary, time window modifies our approach: within 0–6 h one might intervene more readily (particularly if high-risk features) since EVT is proven safe/efficacious in general; in the 6–24 h range, in absence of robust data, management is mostly conservative unless compelling signs of impending infarct expansion exist. Future trials extending criteria to mild strokes will inform this area.

### 4.4 Bridging thrombolysis vs. direct EVT

When deciding on EVT for a low-NIHSS LVO patient, another tactical question is whether to administer IV thrombolysis first (if within 4.5 h window) or proceed directly to thrombectomy. In other words, what is the role of “bridging therapy” in this scenario? Several studies have specifically compared IVT + EVT (bridging) versus IVT alone in minor stroke LVO patients. Interestingly, the data suggest that adding thrombectomy to IVT confers no benefit and might even worsen outcomes in mild strokes. In an international multicenter propensity-matched analysis (1,037 patients) by Schwarz et al., bridging therapy was associated with significantly lower odds of an excellent outcome compared to IV thrombolysis alone (adjusted OR 0.46 (95% CI 0.30–0.72) for mRS 0–1; 0.52 (0.32–0.84) for mRS 0–2; and 1-point mRS shift aOR 1.61 [1.12–2.32]) (53). sICH did not differ significantly (3.3% vs. 1.1%; *p* = 0.082), whereas any hemorrhagic transformation (17.6% vs. 7.3%; *p* < 0.001) and subarachnoid hemorrhage (7.9% vs. 1.5%; *p* = 0.002) were significantly higher with bridging (53). Essentially, doing thrombectomy after tPA did not improve functional results and tended to cause more brain hemorrhages in these mild cases (53). Taken together, these adjusted analyses suggest no functional benefit—and potential bleeding harm—with routine bridging in low-NIHSS anterior-circulation LVO; however, the authors emphasized the need for randomized trials to define optimal practice in this population (53). This result aligns with the earlier finding by Seners et al. that overall outcomes were similar with or without EVT, but sICH risk tripled with bridging therapy (32). The caveat, as noted, was that the impact differed by occlusion site—bridging was potentially beneficial in M1 occlusions but detrimental in M2 occlusions (32). Importantly, center-level procedural heterogeneity may contribute to the observed harm signal with bridging. Lower first-pass effect rates and less frequent balloon-guide catheter use, together with greater device-pass counts/procedure time and differences in first-line technique (stent-retriever vs. direct aspiration vs. combined), have all been linked to variability in reperfusion efficiency and outcomes; such heterogeneity could plausibly increase hemorrhagic transformation when EVT is layered on top of IVT (54).

What about direct EVT without IVT in these patients? This scenario would occur if a patient presents beyond the thrombolysis window or has a contraindication, or if one elects to skip thrombolysis. There is scant data specifically focusing on direct EVT in low-NIHSS strokes. However, one can extrapolate from bridging studies: since IVT appears beneficial (or at least not harmful) in minor LVO and since adding EVT increased hemorrhage, a reasonable inference is that IVT should be given when possible, and EVT can be omitted or deferred unless needed. In practice, if a low-NIHSS LVO patient is taken for thrombectomy, some interventionalists might choose to withhold tPA (to minimize bleeding risk during EVT), but given the evidence above, many would still give tPA as there’s a chance EVT might be aborted if the patient improves or recanalizes spontaneously. Another approach used in some centers is the “drip-and-ship-and-see”: give tPA, transfer for possible thrombectomy, but if on arrival the patient is stable/improved and vessel recanalized on angiography, do not proceed with EVT. On the other hand, if one were absolutely certain that an occlusion needed to be opened (e.g., an ICA occlusion with very poor collaterals in a patient just at NIHSS 5 but looking unstable), some might skip tPA and go straight to EVT to maximize speed and avoid tPA-related hemorrhage. Consistent with the foregoing observation that bridging confers no clear advantage and may increase bleeding, Tu et al. analyzed 903 patients with NIHSS <6 in the Bigdata Observatory Platform for Stroke of China, comparing direct EVT (*n* = 662) with IVT → EVT bridging (*n* = 241). Three-month poor functional outcome and mortality did not differ between groups, whereas symptomatic intracerebral hemorrhage was significantly lower with direct EVT (4.2% vs. 8.3%; *p* = 0.02). These findings met the prespecified non-inferiority criterion for direct EVT and support prioritizing direct EVT—or at least avoiding routine bridging—in minor LVO, particularly when hemorrhagic risk is a concern (55). For minor stroke management, if EVT is planned, omitting tPA could theoretically reduce hemorrhagic complications. However, given that current data question the benefit of EVT itself in this group, the more common scenario is treating medically (with tPA if eligible) and only doing EVT if necessary—effectively a delayed or “selective” thrombectomy strategy rather than planned upfront EVT. In summary, bridging therapy does not appear advantageous in mild LVO stroke and may increase harm (53). If intervention is deemed necessary, one must individualize the choice of giving thrombolysis first versus proceeding directly, weighing bleeding risk, expected EVT delays, and chance of spontaneous improvement.

## 5 Conclusion and future directions

Clinical Recommendations: Based on current evidence, routine endovascular thrombectomy is not recommended for all patients with acute LVO stroke and NIHSS ≤5. Best medical therapy—including IV thrombolysis (if eligible) or antithrombotic therapy—yields generally favorable outcomes in most of these patients, with a substantially lower risk of procedural complications (7). Importantly, randomized evidence specifically enrolling low-NIHSS LVO patients remains limited; consequently, the comparative statements below draw largely on high-quality observational cohorts, matched registries, and retrospective meta-analyses. Across meta-analyses and matched registry studies, rates of 90-day functional independence were similar between EVT and medical therapy; several analyses also noted higher sICH and a possible increase in mortality with EVT, although causality cannot be inferred from non-randomized data (53). These data underscore that an initially mild stroke often remains mild with conservative management. Thus, an initial medical-management-and-observation approach is justified for many low-NIHSS LVO patients. These recommendations should be regarded as provisional and are expected to be refined as forthcoming randomized data become available.

However, management must be individualized. Key factors to consider in decision-making include occlusion site, symptom severity (and whether deficits are disabling), collateral status, clot burden, and patient comorbidities. A reasonable clinical pathway can be proposed:

1. Assess Deficit and Disability: Evaluate NIHSS sub-items—a low total score may hide a disabling deficit (e.g., isolated aphasia or hemianopsia) (56). If the patient has any clearly disabling neurological deficit despite NIHSS≤5, treat aggressively (IVT if eligible, and consider EVT). For non-disabling or very mild symptoms, proceed to step 2.
2. Urgent Vascular Imaging: Confirm the presence of LVO (CTA/MRA). If no LVO, treat as per minor stroke (usually medical management). If LVO is present, evaluate occlusion location and collaterals: Proximal occlusion (ICA or M1) and/or poor collaterals: high risk for deterioration—lean towards reperfusion therapy. Administer IV thrombolysis if within window. Plan for EVT if safe and feasible, especially if any subtle progression in symptoms or imaging shows large at-risk territory. Some may perform immediate EVT in these high-risk cases, while others may observe briefly for changes—but have a low threshold to intervene. Distal occlusion (M2 or beyond) and good collaterals: likely lower risk—lean towards conservative management. Give IVT if eligible [particularly for M2 occlusions, as tPA often suffices (32)], but otherwise treat medically (aspirin or DAPT) and observe. Do not rush to angio unless clinical worsening occurs.
3. Monitor Closely for END: Admit to a stroke unit or ICU for neurological checks every hour initially. If any early neurological deterioration occurs (e.g., NIHSS increases by ≥4, or new deficits), escalate to rescue EVT immediately (if within a feasible time window and patient still has salvageable tissue). The decision for rescue can be made even up to 24 h if there is clinical worsening and imaging shows an occluded vessel with penumbra. Conversely, if the patient remains stable or improves, continue medical management.
4. Reassess at 24 Hours: Perform repeat imaging to confirm recanalization status and infarct progression. An MRI or CTA can be done at 24 h. If the vessel remains occluded but the patient is still doing well, invasive intervention at that point is usually not pursued (since they have proven to have a benign course, though a late elective EVT could be considered in rare cases of persistent occlusion with high risk of secondary stroke). If the patient worsened overnight (missed early signs), late EVT could be considered on a case-by-case basis (with appropriate imaging selection).

This pathway essentially embodies a “selective EVT” strategy: treat all with best medical therapy (and IV tPA if indicated), then selectively deploy EVT for those with high-risk features or who demonstrate deterioration. Such an approach is supported by available evidence and expert consensus (33). Table 2 provides an overview of major studies informing these recommendations, highlighting how outcomes compare between EVT and medical management in different subgroups.

**Table 2:** Select studies comparing EVT vs. medical therapy in low-NIHSS LVO stroke.

Study (Year)	Population (LVO with NIHSS≤5)	Comparison groups	Key findings	Conclusion/notes
**Matusevicius et al., 2022** (6)	544 matched pts. (GSR-ET registry vs. SITS registry); anterior & posterior LVO	EVT (± IVT) vs. IVT-alone. IVT (no EVT) was independent predictor of good outcome	3-mo good outcome: 77.0% vs. 82.9% (*p* = 0.12); sICH: 8.8% vs. 12.5% (n.s.) (6). IVT (no EVT) was independent predictor of good outcome (6).	No significant outcome difference between EVT and medical therapy. Suggested IVT is as effective as EVT in minor LVO (6).
**Schwarz et al., 2023** (53)	624 matched pts. (multi-center; anterior circulation LVO)	IVT + EVT (bridging) vs. IVT-alone	Bridging therapy had lower odds of mRS 0–1 (aOR 0.46) and 0–2 (aOR 0.52) vs. IVT (53). sICH: 3.3% vs. 1.1% (*p* = 0.08); any ICH significantly higher with EVT (53).	EVT added to IVT did not improve outcomes in mild LVO; trend toward more hemorrhagic complications (53). Suggests no benefit to routine bridging in NIHSS≤5.
**Alexandre et al., 2023** (33)	200 pts. (multicenter; *isolated M2* occlusion only)	Immediate EVT vs. Medical (+ rescue EVT if needed)	No significant difference in 90-day mRS 0–1 or safety between upfront EVT and medical management (33). ~25% of medical group received rescue EVT (for END).	In isolated M2 occlusion with mild stroke, upfront EVT showed no benefit over best medical therapy. Reasonable to manage medically first and perform EVT only if END occurs (33).
**Zhao et al., 2024** (7)	22 studies (4,985 pts., 2015–2023) spanning various populations (anterior LVO, NIHSS≤5)	EVT (± IVT) vs. Medical (± IVT)	No overall difference in 90-day mRS 0–1 or 0–2 between EVT vs. medical (7). EVT had higher 90-day mortality (OR 1.84) and higher sICH risk (OR 3.36) (7). Subgroup: elevated hemorrhage risk with EVT seen for both proximal and distal occlusions (7).	No clear efficacy advantage of EVT over medical therapy on average in mild strokes; observational safety signals warrant caution and support the need for randomized trials (7).
**Yedavalli et al., 2023** (10)	46 pts. (2 centers; M1 or proximal M2 occlusion)	EVT vs. Medical management	90-day median mRS: 1 (EVT) vs. 3 (Med), *p* < 0.001; higher rate of excellent outcome and NIHSS improvement with EVT (10).	Small retrospective study suggesting EVT can improve outcomes in carefully selected minor stroke patients (10). Supports treatment in some cases, but limited sample (*n* = 11 EVT).

*Matched observational studies (NIHSS ≤5). “Good outcome” = mRS 0–2 (except Yedavalli et al., 2023). END = NIHSS increase ≥4 within 24–48 h. Bold values = *p* < 0.05.

As the table shows, the preponderance of evidence currently leans towards conservative management with selective intervention. For most low-NIHSS LVO patients, best medical therapy (including IV thrombolysis when appropriate) is the initial strategy, and EVT is reserved for those with high-risk occlusions or early clinical worsening. This approach minimizes unnecessary invasive procedures and hemorrhagic complications (7). While still allowing patients who declare themselves (by deteriorating) to receive thrombectomy rescue (7).

### 5.1 Future research

Completed randomized data specific to NIHSS ≤5 LVO remain sparse; despite insights from observational studies, the field urgently needs prospective randomized trials to provide higher-quality evidence. Fortunately, several trials are underway or recently completed. Notably, the ENDOLOW trial (NCT04167527) is a randomized study specifically evaluating EVT versus medical management in patients with NIHSS <6 and LVO—its results (expected soon) should directly inform this debate. Other trials like MOST (Minor Stroke Therapy) are also aiming to enroll minor stroke LVO patients. Key questions include: Can we identify which mild LVO patients benefit from immediate thrombectomy? Is there a role for advanced imaging selection (perfusion/diffusion mismatch) in these low-NIHSS patients? How effective is a “watchful waiting” strategy in an RCT setting (some trials have arms that allow delayed EVT if deterioration occurs)? Additionally, research into biomarkers and scoring systems for END risk stratification is ongoing—for instance, validating the aforementioned END prediction scores in prospective cohorts. Ongoing randomized trials directly comparing EVT with medical management in low-NIHSS LVO are summarized in Table 3, including key inclusion criteria and primary outcomes. Accordingly, conclusions in this review should be interpreted as interim and may evolve as these randomized results are reported.

**Table 3:** Ongoing randomized trials comparing endovascular thrombectomy (EVT) vs. medical management in low-NIHSS (≤5) large-vessel occlusion stroke: key inclusion criteria and primary outcomes.

Trial (NCT)	Key inclusion (abridged)	Arms	Primary outcome	Status
ENDOLOW (NCT04167527)	Anterior-circulation LVO (intracranial ICA/M1 or “M1-like” M2); NIHSS 0–5; treat ≤8 h; imaging limits (e.g., ASPECTS ≥6); age ≥18.	Immediate mechanical thrombectomy vs. initial medical management (guideline care incl. IVT if eligible).	90-day mRS distribution (ordinal shift).	Active (not recruiting); est. completion 2025 (63).
MOSTE (NCT03796468)	Minor stroke with anterior-circulation LVO (intracranial ICA/M1 ± proximal M2); NIHSS ≤5; ≤23 h LSW; ASPECTS ≥6; age ≥18.	Immediate MT + best medical therapy vs. best medical therapy alone (rescue MT if deterioration).	mRS 0–1 at 90 days.	Recruiting; est. completion 2025 (64).
STEP platform (Domain A) (NCT06289985)	Platform trial; low-NIHSS stratum: intracranial ICA/M1 LVO, NIHSS 0–5; ≤24 h; pre-stroke mRS 0–2.	EVT vs. Medical management (domain-specific randomization; adaptive).	90-day mRS (platform primary); safety incl. sICH.	Recruiting / launched 2025 (65).

LVO, large-vessel occlusion; EVT, endovascular thrombectomy; IVT, intravenous thrombolysis; mRS, modified Rankin Scale; ICA, internal carotid artery; M1/M2, first/s MCA segment. Trial status and details were extracted from public registries.

Another future direction is refining the definition of “minor stroke” in the context of LVO. NIHSS alone may be too blunt; using a combination of NIHSS and deficit disabling status could better select patients for trials. Also, patient-centered outcomes (quality of life, cognitive outcomes) should be studied—even if a patient has an NIHSS 3, subtle cognitive deficits from an untreated LVO infarct might impact long-term life, which might be mitigated by reperfusion. Most studies in this field rely on 90-day modified Rankin Scale (mRS) as the primary endpoint; however, the mRS has known ceiling effects and correlates only modestly with cognition and health-related quality of life, especially after mild strokes. Substantial proportions of “excellent mRS” survivors still experience post-stroke cognitive impairment and patient-important neuropsychiatric sequelae such as depression, which are not captured by the mRS. Accordingly, when interpreting evidence in low-NIHSS LVO, we acknowledge that mRS-based analyses may underestimate treatment effects on cognition, mood, fatigue, and return-to-work. Future trials and clinical pathways should include brief cognitive screening (e.g., MoCA) and patient-reported outcomes (e.g., Neuro-QoL/PROMIS or SIS-16) alongside mRS (57).

Additionally, the impact of END definitions on reported rates and associations. Reported END rates vary widely because definitions differ across studies. Lower NIHSS thresholds (e.g., ≥2 points) and longer observation windows (24–48 h or 7 days) systematically increase END frequency compared with ≥4 points within 24 h; conversely, very-early ND (within 1 h post-IVT) captures a narrower, treatment-proximal subset and yields lower absolute rates. These definitional choices also influence effect estimates (e.g., END as a predictor of 90-day outcomes), with broader definitions generally increasing sensitivity but potentially diluting specificity for poor outcomes. Therefore, differences in END definitions represent a nontrivial source of heterogeneity in pooled analyses of low-NIHSS LVO (58).

In conclusion, the management of LVO strokes with low NIHSS remains a nuanced and debated topic. Current evidence favors an individualized approach: treat with thrombolysis and medical therapy initially in most cases, and pursue EVT selectively based on occlusion characteristics or clinical course. For now, there is no one-size-fits-all answer, and clinicians must weigh the immediate mild status against the potential for deterioration on a case-by-case basis. Ongoing trials and future research will hopefully resolve the controversy by identifying which patients truly benefit from early intervention and which are safely managed without thrombectomy. Until then, a balanced approach that prioritizes patient safety while remaining vigilant for signs of worsening is the prudent strategy (6).

## 6 Critical appraisal of the evidence

The current evidence base is dominated by observational cohorts and study-level meta-analyses; randomized evidence dedicated to low-NIHSS populations is still lacking. The largest meta-analysis restricted to anterior-circulation low-NIHSS (NIHSS ≤5) reported no improvement in 90-day functional outcomes with EVT versus best medical therapy and a higher risk of sICH, with most included studies being nonrandomized and substantial between-study heterogeneity (59).

Two biases likely shape observed effects. Confounding by indication: patients triaged to EVT more often have disabling deficits, larger clot burden, poorer collaterals, or evolving END, worsening outcomes independent of treatment and making EVT appear less effective/harmful in crude analyses (60). Crossover/rescue bias (BMT → EVT upon deterioration) can dilute any true differences in intention-to-treat–like comparisons.

Given these nonrandomized designs and inconsistency, the certainty of evidence for functional benefit is low to very low by GRADE (downgraded for risk of bias, inconsistency/heterogeneity, indirectness), whereas the signal for increased sICH with EVT is more consistent but still limited by residual confounding (61). Dedicated RCTs in low-NIHSS populations are needed.
